# Pan-genome sequence analysis using Panseq: an online tool for the rapid analysis of core and accessory genomic regions

**DOI:** 10.1186/1471-2105-11-461

**Published:** 2010-09-15

**Authors:** Chad Laing, Cody Buchanan, Eduardo N Taboada, Yongxiang Zhang, Andrew Kropinski, Andre Villegas, James E Thomas, Victor PJ Gannon

**Affiliations:** 1 Laboratory for Foodborne Zoonoses, Public Health Agency of Canada, Lethbridge, AB, Canada; 2 Laboratory for Foodborne Zoonoses, Public Health Agency of Canada, Guelph, ON, Canada; 3 Faculty of Biological Sciences, University of Lethbridge, Lethbridge, AB, Canada

## Abstract

**Background:**

The pan-genome of a bacterial species consists of a core and an accessory gene pool. The accessory genome is thought to be an important source of genetic variability in bacterial populations and is gained through lateral gene transfer, allowing subpopulations of bacteria to better adapt to specific niches. Low-cost and high-throughput sequencing platforms have created an exponential increase in genome sequence data and an opportunity to study the pan-genomes of many bacterial species. In this study, we describe a new online pan-genome sequence analysis program, Panseq.

**Results:**

Panseq was used to identify *Escherichia coli *O157:H7 and *E. coli *K-12 genomic islands. Within a population of 60 *E. coli *O157:H7 strains, the existence of 65 accessory genomic regions identified by Panseq analysis was confirmed by PCR. The accessory genome and binary presence/absence data, and core genome and single nucleotide polymorphisms (SNPs) of six *L. monocytogenes *strains were extracted with Panseq and hierarchically clustered and visualized. The nucleotide core and binary accessory data were also used to construct maximum parsimony (MP) trees, which were compared to the MP tree generated by multi-locus sequence typing (MLST). The topology of the accessory and core trees was identical but differed from the tree produced using seven MLST loci. The Loci Selector module found the most variable and discriminatory combinations of four loci within a 100 loci set among 10 strains in 1 s, compared to the 449 s required to exhaustively search for all possible combinations; it also found the most discriminatory 20 loci from a 96 loci *E. coli *O157:H7 SNP dataset.

**Conclusion:**

Panseq determines the core and accessory regions among a collection of genomic sequences based on user-defined parameters. It readily extracts regions unique to a genome or group of genomes, identifies SNPs within shared core genomic regions, constructs files for use in phylogeny programs based on both the presence/absence of accessory regions and SNPs within core regions and produces a graphical overview of the output. Panseq also includes a loci selector that calculates the most variable and discriminatory loci among sets of accessory loci or core gene SNPs.

**Availability:**

Panseq is freely available online at http://76.70.11.198/panseq. Panseq is written in Perl.

## Background

The field of genomics has blossomed as a result of the fast rate of whole-genome sequence data acquisition. The pace of genome data growth continues to increase as the cost to acquire the data continues to decrease. This has been led in large part by massively parallel sequencing platforms such as the 454 Genome Sequencer FLX (Roche Applied Science), the Illumina (Solexa) Genome Analyzer and the ABI SOLiD System (Applied Biosystems), which generate tens of millions of base pairs of information in short reads 30 to several hundred base pairs in length [1,2]. These reads must be combined into large contiguous DNA sequences by dedicated software such as Newbler (Roche) and MAQ [2]. Although these "contigs" can stretch into the megabase-pair (Mb) range, the sequencing of an entire organism by any one of these techniques invariably leaves gaps in the reassembled sequence [3]. The finishing of a sequence requires gap-closure by sequencing of PCR products and the resolution of sequencing errors. Sequencing efforts are primarily driven by the discovery of novel genes and, as gap closure is time-consuming and expensive, many researchers now use un-finished draft sequences of genomes in their analyses [4].

Tettelin et al. [5] used the term "pan-genome" to refer to the full complement of genes within a bacterial species. Comprising the pan-genome are the core complement of genes common to all members of a species and a dispensable or accessory genome that is present in at least one but not all members of a species. As more whole-genome sequences of a species or group within a species become available, the size of the pan-genome of that species or group will usually increase, due to an increase in the number of accessory genes. Based on mathematical models, it is predicted that new genes will be discovered within the pan-genome of many free-living bacterial species even after hundreds or possibly thousands of complete genome sequences have been characterized [6]. While originally applied to an entire species, any group of related strains can be said to contain a "core" and "accessory" set of genes. As such, tools that extract the new pieces of information from an extremely large pool of data and that can be used to determine the pan-genome and its distribution among strains will be invaluable in the study of genotypic and phenotypic traits in bacterial populations.

Regardless of whether an investigator uses a draft or finished sequence, software tools able to efficiently extract relevant information are critical. A number of programs have been designed to assist with the analysis of DNA sequence data. These include programs designed for multiple sequence alignments such as CLUSTAL W [7], T-COFFEE [8] and MUSCLE [9]; programs for local sequence comparison including FASTA [10] and BLAST [11]; and programs designed for whole-genome comparisons such as MUMmer [12], MAUVE [13] and MISHIMA [14]. Pre-computed alignments of completed genomes are available online from Web-ACT [15] (which also allows up to five user-submitted comparisons), the map-viewer and gMap database features from the National Center for Biotechnology Information [16], the MOSAIC web-server [17] and the prokaryotic gene-order database PSAT [18].

Programs designed specifically to find mobile genomic islands as opposed to regions of differing sequence between or among genomes using comparative genomic approaches include MobilomeFINDER [19] and IslandPick [20]. Three programs offering *in silico *subtractive hybridization among genomes using BLAST are available: FindTarget [21], mGenomeSubtractor [22] and nWayComp [23]. See Additional File 1 for a comparison of features among many of these programs.

Despite the myriad program options available, there is no comprehensive package for pan-genome analysis. Prior to the advent of the current generation of sequencing platforms, the number of genome sequences available for intra-species comparative genomic analysis and for the determination of "accessory" genes was limiting; consequently, tools specifically designed for the analysis of the pan-genome have not been available.

Most studies have used one or two reference genomes, which include the core elements but are missing much of the accessory genome of the species in study. In the laboratory, selective subtractive hybridization [24,25] and population surveys using microarray comparative genomics [26,27] have been used to examine/define accessory genome content. With the current explosion in availability of whole-genome draft sequences, it would be highly desirable to exploit this information through *in silico *analysis to separate the novel accessory component of the genome from previously identified core sequence without the requirement for a finished assembly. Identifying novel accessory sequences in this way has application in characterizing novel metabolic pathways, virulence attributes [28], and molecular fingerprinting targets useful in epidemiological and population genetic studies [29]. Finally, both the core and accessory genomes can be helpful in elucidating the evolutionary history of organisms [30].

In this study we describe a pan-genome sequence analysis program, Panseq, that extracts novel regions with respect to a sequence or group of sequences, determines the core and accessory regions of sequences based on sequence identity and segmentation length parameters, creates files based on the core and accessory genome for use in phylogeny programs and determines the most discriminatory and variable set of loci from a dataset. We also report the validation of Panseq outputs using PCR experiments and comparisons to previously published genome sequence analyses.

### Implementation

The pan-genome sequence analysis program (Panseq) was written in Perl with BioPerl [31] modules and is available at http://76.70.11.198/panseq. As a web-server it is platform independent and makes use of the NCBI Genbank database for pre-existing nucleotide FASTA files. The Perl scripts are available for standalone use by contacting the author.

### Novel Region Finder (NRF)

The NRF module compares an input sequence(s) to a database of sequence(s), and contiguous regions not present in the database but present in a user-defined combination of input sequences are extracted. This is accomplished using the MUMmer alignment program [12] with the "novel" sequences extracted into a file with sequence location information added to the FASTA header. This process is iterative, adding the initial sequence examined to the database before examining the second sequence, and so on until all sequences have been examined. Because the algorithm examines all matches given user-specified criteria, regions of high sequence identity will also be matched independent of their order (non-syntenic). A summary file indicating the size distribution of fragments and total number of novel nucleotides is created, as well as a graphical representation of the novel regions distributed among all input sequences in scalable vector graphic (SVG) form. Default Nucmer parameters used by the NRF module are: b:200, c:50, d:0.12, g:100 and l:20; where according to the MUMmer manual http://mummer.sourceforge.net/manual/#nucmer, b: the distance an alignment will extend poor scoring regions; c: the minimum cluster length; d: the maximum diagonal difference (diagonal difference/match separation); g: the maximum gap between two adjacent matches in a cluster; l: the minimum length of an exact match.

### Core and Accessory Genome Finder (CAGF)

The CAGF module considers for the purposes of analyses the "pan-genome" to be comprised of sequences selected as input to the program. Panseq initiates using a single sequence file as a seed to which all other sequences are compared using MUMmer. If a segment greater than the 'Minimum Sequence Size' is found in a sequence other than the seed, that segment is added to the "pan-genome". This newly-added-to "pan-genome" is used as the reference for subsequent comparisons and the process continues iteratively until all sequences have been examined. Panseq next fragments the entire pan-genome into segments of user-defined length and determines the presence or absence of each of these fragments in each of the original sequences based on the percent sequence identity cutoff using the BLASTn algorithm [11], with the following default parameters: blastall -p blastn -W 11, -b (2*<number of input sequences>) -v (2*<number of input sequences>) -e 0.001, -F F. Fragments above the cutoff found in every original sequence are considered part of the "core" genome, while fragments below the cutoff in at least one strain are considered part of the "accessory" genome.

The core genome for each input sequence is concatenated into a single sequence and a multiple sequence alignment is produced. The accessory genome is reported in a tab-delimited table, where binary (0 for absence, 1 for presence) data indicate the state of each fragment in the original sequences. A NEXUS formatted file [32] for both the accessory and core genomes are output for use in downstream phylogenetic applications. Panseq also produces a SNP file containing core segments with sequence variability; a tabular file listing each SNP, its position, and value among each original sequence; "core" and "accessory" genomes output to separate FASTA files; and a scalable vector graphic depicting the pan-genome and its presence/absence among all of the original input sequences.

### Loci Selector (LS)

The LS module constructs loci sets that are maximized with respect to the unique number of fingerprints produced among the input sequences as well as the discriminatory power of the loci among the input sequences. The LS module iteratively builds the final loci set, in the following steps, given a tab-delimited table with loci names in the first column, sequence names in the first row, and single character data filling the matrix:

(1) Each potential available locus is evaluated for the number of unique fingerprints that would result from its addition to the final loci set. All loci that would generate the maximum number of unique fingerprints in this respect are evaluated in step (2).

(2) All loci from step (1) are evaluated for their discriminatory power among the sequences, which is given as points of discrimination (POD). The POD for a locus is calculated as follows.

A listing of all possible pair-wise comparisons is constructed; for example, if the input table consisted of three sequences, A, B and C, the list would consist of A-B, A-C and B-C. Next, it is determined whether or not the sequences in each pair-wise comparison contain the same single character denoting the locus state. If they do, a value of 0 is assigned; if they differ a value of 1 is assigned. The POD is then the summation of all pair-wise comparisons that differ for that locus. With our previous example, if A-B = 1, A-C = 1 and B-C = 0, the POD for that locus would be 2.

(3) The locus with the highest value from step (2) is selected for addition to the final loci set and removed from the pool of candidate loci. If two or more loci tie in value, one is randomly selected. If all possible unique fingerprints have been found, the algorithm continues with (4); if additional unique fingerprints are possible, the algorithm continues with (5).

(4) Sequence pairs for which the allele of the locus chosen in (3) differ are excluded from the analysis. This ensures loci that differ between other pairs of strains are preferentially considered. Consider our A, B and C example with pair-wise comparisons of A-B = 1, A-C = 1 and B-C = 0. In the case of this locus being chosen, the sequence pairs A-B and A-C would be temporarily removed from the analysis ("masked"), leaving only loci that differed between B-C as viable options.

(5) Once a locus has been chosen:

a) the specified number of loci has been reached (all unique fingerprints in the case of 'best') and the algorithm terminates; or

b) the specified number of loci has not been reached and there are remaining fingerprints possible, or sequence pairs for which differences exist. The algorithm returns to (1); or

c) there are no remaining fingerprints possible and no sequence pairs for which differences exist. At such time, all sequence pairs are again considered part of the analysis ("unmasked"). If no differences among any sequence pairs exist at this point, the algorithm terminates; if differences remain, the algorithm returns to (1).

## Results and Discussion

We have used a number of examples to highlight the functionality of Panseq, many of which could be carried forward as complete studies of their own; however, our intention is to demonstrate that Panseq is capable of finding and extracting useful data from sequences, which can be used as the basis for hypothesis generation and future investigations.

### (1) Novel Region Finder (NRF) Module

Alignment programs are capable of finding regions of similarity between sequences, and regions of uniqueness can be inferred from the gaps between areas with high sequence similarity. However, a number of steps are required using alignment programs to identify genomic regions that are unique with respect to other sequence(s). These steps include the location of the sequence coordinates for each sequence of interest and the subsequent location of the corresponding sequence in a sequence editor. Panseq automates this process, creating FASTA files of all unique regions of a given sequence or sequences as well as presenting a graphical overview of the locations of the novel regions, based on the results of sequence comparisons made using the MUMmer algorithm. MUMmer was chosen as the sequence alignment engine because of its use of suffix-trees, which allow it to perform operations up to 100 times faster than similar alignment programs in whole-genome comparisons [12].

Determining putative functions for the regions identified by the NRF module can be accomplished by comparing the translated nucleotide sequences to known protein sequences using the Panseq links to NCBI BLASTx [11] or the UniProt database [33]. This linking allows the genomic information to be easily queried and is a logical first step in connecting genotype to phenotype in comparative genomics analyses.

#### The NRF Module in Genomic Island Identification

Novel regions have the potential to affect virulence and niche specificity of pathogenic microorganisms. In 2000, Perna et al. [34] published the complete genomic sequence of the pathogenic *E. coli *O157:H7 strain EDL933 and compared it to the previously sequenced, non-pathogenic laboratory *E. coli *K12 strain MG1655. Genomic regions found in *E. coli *O157:H7 EDL933 but not in K12 were called "O-Islands" and genomic regions present in K12 but not EDL933 were termed "K-Islands". Perna et al. found 177 O-Islands greater than 50 bp in *E. coli *O157:H7 EDL933, constituting 1.34 Mb and 234 K-Islands greater than 50 bp in the K12 strain MG1655, representing 0.53 Mb. They determined the presence of these islands using a custom modification of the MUMmer program [12] and found that many of the genomic regions in strain EDL933 were bacteriophage-related and suggested that they may play a role in the virulence of the organism.

Using Panseq, we re-analyzed the genome sequence data used in these experiments with the NRF module, checking "Unique among the sequences selected" to generate both sets of genomic islands in a single step. Perna et al. [34] defined islands as regions with less than 90% sequence identity over 90% of the sequence length. We found that genomic islands identified in this previous analyses can extend into conserved regions, combining multiple islands into a single contiguous sequence that is interspersed with regions of high sequence identity. Similarly, genomic islands may be misclassified as core regions simply because of heterogeneous composition with interspersed core and accessory sequences in regions of high heterogeneity. Panseq uses parameters optimized for high-resolution comparison of genomic sequences and we found that it more stringently defined islands unique to a genome sequence.

With default program settings (Nucmer values of b:200, c:50, d:0.12, g:100, l:20) and a minimum novel region size of 50 bp, Panseq identified 214 K- and 304 O-Islands, compared with the 234 K- and 177 O-Islands of Perna et al. [34]; a detailed comparison of the findings can be found in Additional File 2. As a result of differences in the stringency between the two methods, present but heterogeneous islands such as K-Island #4 were not identified as islands by Panseq; K-island #4 matches at 90% sequence identity for 84 bp at positions 111429 - 11511 in K-12 and 116035 - 116118 in EDL933. This represents an example of modest differences in core sequence rather than of "novel" regions that comprise the accessory genome (see also below the discussion of identity thresholds). Additionally, islands identified by Perna et al. with interspersed regions of high sequence identity were split and refined into separate islands by Panseq; for example O-island #7 was split into four unique islands, eliminating the regions of high sequence identity. These results demonstrate that Panseq can correctly identify and rapidly extract novel genomic sequences.

Two-way comparisons similar to those used for *E. coli *strains K-12 and EDL933 have been used to identify genetic attributes which confer distinct phenotypes on many other taxonomically related strain pairs. This type of analysis has allowed researchers to identify elements unique to each strain and suggest putative functions for these elements in the life cycles of the respective organisms, their virulence and their ability to survive in different niches. Examples of such studies include the identification of genetic differences between the human-restricted *Salmonella enterica *subspecies *enterica *serovar Typhi, and *Salmonella enterica *subspecies *enterica *serovar Typhimurium, which is a murine pathogen but is not host-restricted [35]; and the differences between the host-restricted causative agent of plague, *Yersinia pestis *and the enteric pathogen *Yersinia pseudotuberculosis *[36]. While these types of two strain comparison studies have been extremely insightful, studies using programs such as Panseq will greatly facilitate these comparisons and also allow comparisons between multi-strain groups.

#### The NRF Module in Multiple Sequence Comparisons

**Figure 1:**
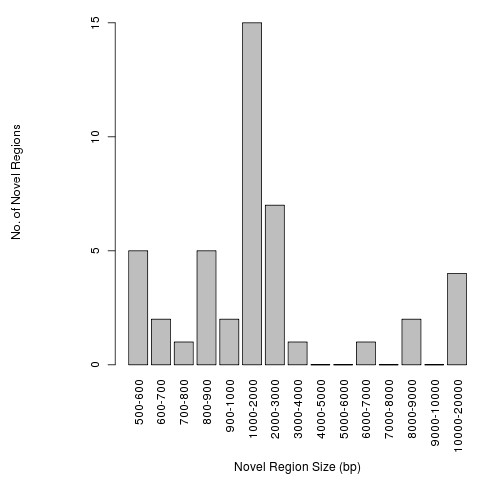
**Size distribution of *Listeria monocytogenes *strains F6900 and 10403S novel regions**. The size distribution of regions novel to one or both *Listeria monocytogenes *strains F6900 and 10403S with respect to the four *L. monocytogenes *strains Clip81459, EGD-e, HCC23 and 4bF2365. The minimum extracted novel region size was 500 bp.

To demonstrate a comparison of recently sequenced genomes to previously completed "reference" genomic sequences, we used the two draft genome sequences of *Listeria monocytogenes *F6900 and 10403S and compared them to the four complete *L. monocytogenes *genomes in Genbank: Clip81459, EGD-e, HCC23 and 4bF2365 (Table 1). The NRF module found 45 novel regions ≥500 bp, constituting 126858 bp of genomic DNA not present in the four reference *L. monocytogenes *genomes. The size distribution of the novel regions from the output file is presented in Figure 1. In addition to the summary file, the novel regions are output in FASTA format with sequence location and size information found in the header. These files are suitable for additional bioinformatic or phylogenetic analysis. In addition to the FASTA file, an SVG graphics file showing the location of the novel regions in each strain is optionally provided. As can be seen in Figure 1, the greatest number of novel regions was found in the 1000 -2000 bp range, with 38 of the 45 regions less than 4000 bp in size.

**Table 1:** The 6 *Listeria monocytogenes *genomic sequences analyzed, with RefSeq accession numbers and genomic sequence status.

RefSeq Accession No.	Strain	Genome Status
NC_002973	4b F2365	Complete
NC_003210	EGD-e	Complete
NC_011660	HCC23	Complete
NC_012488	Clip81459	Complete
NZ_AARU02	F6900	Draft
NZ_AARZ02	10403S	Draft

#### Experimental Confirmation of Outputs from the NRF Module

We wished to experimentally confirm the uniqueness of the novel regions extracted by Panseq. To do this, we determined genomic regions present in at least one of 14 *E. coli *O157:H7 whole genome sequences (Table 2) but absent from the two O157:H7 reference genomes EDL933 and Sakai. We designed primers targeting 65 of these novel regions and examined the distribution of the regions among a population of 60 *E. coli *O157:H7 strains. All of the primer pairs generated amplicons in at least one but not all of the *E. coli *O157:H7 strains other than EDL933 and Sakai, and all failed to amplify DNA from *E. coli *O157 EDL933 and Sakai, as predicted by Panseq (data not shown). This experimentally demonstrated that the sequences identified by Panseq represent novel accessory regions not found in the two reference *E. coli *O157:H7 genomes.

**Table 2:** The 14 *E. coli *O157:H7 strains compared by Panseq to the *E. coli *O157:H7 reference strains EDL933 and Sakai for novel accessory genomic regions.

RefSeq Accession No.	Strain
NZ_ABJT00000000	*Escherichia coli *O157:H7 str. EC4024
NZ_ABHM00000000	*Escherichia coli *O157:H7 str. EC4042
NZ_ABHL00000000	*Escherichia coli *O157:H7 str. EC4045
NZ_ABHQ00000000	*Escherichia coli *O157:H7 str. EC4076
NZ_ABHP00000000	*Escherichia coli *O157:H7 str. EC4113
NZ_ABHO00000000	*Escherichia coli *O157:H7 str. EC4196
NZ_ABHK00000000	*Escherichia coli *O157:H7 str. EC4206
NZ_ABHR00000000	*Escherichia coli *O157:H7 str. EC4401
NZ_ABHS00000000	*Escherichia coli *O157:H7 str. EC4486
NZ_ABHT00000000	*Escherichia coli *O157:H7 str. EC4501
NZ_ABHW00000000	*Escherichia coli *O157:H7 str. EC508
NZ_ABHU00000000	*Escherichia coli *O157:H7 str. EC869
NZ_ABKY00000000	*Escherichia coli *O157:H7 str. TW14588
NC_011353.1	*Escherichia coli *O157:H7 str. EC4115

### (2) Core/Accessory Genome Finder (CAGF) Module

**Figure 2:**
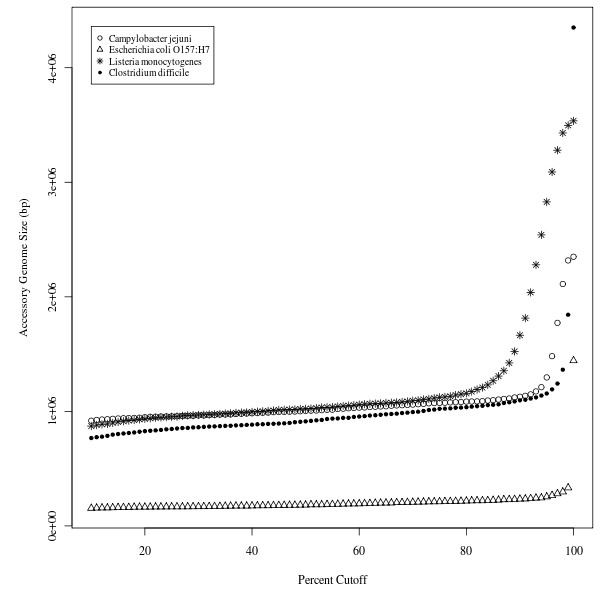
**Variation in the size of the accessory genome with respect to sequence identity cutoff**. The size of the accessory genomes for groups of *Listeria monocytogenes *strains (F6900, 10403S, Clip81459, EGD-e, HCC23 and 4bF2365); *E. coli *O157:H7 strains (EDL933, Sakai, EC4115, TW14539); *Clostridium difficile *strains (630, CD196, R20291, BI9); *Campylobacter jejuni *strains (RM1221, 81-176, 81116, NCTC 11168, 269.97) over sequence identity cutoff values of 10 - 100%. Genomes were fragmented into 500 bp segments.

The Panseq CAGF module uses a combination of fragmentation and sequence identity thresholding to define accessory and core genomic regions. To determine the effect of the sequence identity cutoff on core/accessory genome size, we examined groups of *L. monocytogenes, E. coli *O157:H7, *Clostridium difficile *and *C. jejuni *genomes with a fragmentation size of 500 bp (~ half the size of an average gene) over a range of sequence identity cutoffs (Figure 2). The size of the estimated accessory genome increased as the sequence identity threshold was raised, and the size of the estimated core genome decreased proportionally; however, this increase was observed to have two distinct phases: an initial linear growth in accessory genome size that was followed by an exponential increase in accessory genome size. The transition between the two stages occurred in the 80 - 90% sequence identity cutoff range for each species, suggesting that with values below 80% Panseq primarily identified accessory genome segments that were variably absent or present whereas above this threshold Panseq identified core genome segments with sequence heterogeneity.

#### The CAGF Module in SNP Analysis of the Core Genome

Accessing the core genome is important for phylogenetic studies, which have used "core" gene concatenates ranging in size from a few genes in multi-locus sequence typing (MLST) schemes for *Campylobacter jejuni *[37] and *L. monocytogenes *[38] to all known "core" genes in *E. coli *O157:H7 [39]. In addition to offering the best available data for assessing the phylogenetic reconstruction of the evolutionary history of an organism, a small number of single nucleotide polymorphisms (SNPs) can be useful in defining clusters of epidemiologically related strains [40-42].

**Figure 3:**
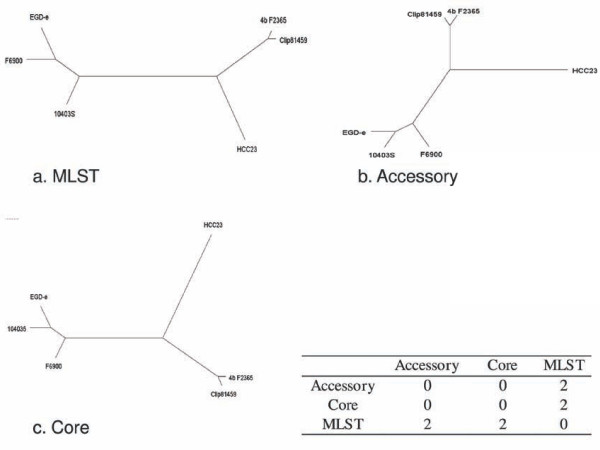
**Maximum parsimony trees generated from MLST, accessory and core genome data from *Listeria monocytogenes *strains**. The maximum parsimony (MP) trees generated for the six *Listeria monocytogenes *strains F6900, 10403S, Clip81459, EGD-e, HCC23 and 4bF2365 using a) multi-locus sequence typing as described by Nightingale et al., 2005 [38]; b) the binary presence/absence data of the accessory genome found using the Panseq Core/Accessory Genome Analysis module with fragmentation size of 500 bp and sequence identity cutoff value of 85%; c) the aligned core genome found with the parameters of b). MP trees were created using Phylip v3.69 [43] with the PARS function. The inset table depicts the symmetrical tree distances between each pair of trees, calculated using the TREEDIST function of Phylip.

We analyzed the six *L. monocytogenes *strains in Table 1 with the CAGF module of Panseq, using a fragmentation size of 500 bp and a sequence identity cutoff of 85%. The resulting concatenated core data, which include all conserved nucleotides and SNPs, were used to construct a maximum parsimony (MP) tree using Phylip v3.69 [43]. A MP tree was also generated *in silico *for the same six strains using the *L. monocytogenes *MLST protocol outlined by Nightingale et al. [38], for comparative purposes (Figure 3). The symmetric distance between the two trees shows that the overall topology of the trees differ between that created from MLST data and that based on the entire concatenated core. This is likely due to the fact that the MLST protocol only considers seven genes, where disproportionate variation among these few loci and the relative paucity of loci compared to that of the entire core genome can reduce the ability of this method to capture the overall relationships among strains. With the continued increase in sequencing throughput, settling for rough approximations of true tree topologies may no longer be necessary.

#### The CAGF Module in the Analysis of the Accessory Genome

While SNP analysis has proven to be an extremely useful tool, the ideal reconstruction of an evolutionary history would take into account not only the heterogeneity among all core genomic regions, but also the presence or absence of regions in the accessory gene pool, especially since the accessory genes can directly affect phenotype (e.g. niche specificity, antimicrobial resistance, virulence, etc.).

Traditional methods of estimating phylogenies rely on SNPs within the core genome, but it has been shown for *C. jejuni *[44], *E. coli *O157:H7 [45] and *Streptococcus pneumoniae *[46] that the distribution of accessory genes provides a very similar overall tree topology to methods based on variability in the core genome. Although variation in the core genome among a small number of loci may be sufficient for identifying clusters of related strains, discrimination among strains is often more difficult because there is less variation and fewer phylogenetically informative loci, leading to fewer genotypes. Accessory genome content, which can be highly variably among strains, appears in many cases to be consistent with phylogenetic analyses of core genes and as a result of greater variability provides a higher degree of discrimination among strains. Analysis of accessory gene content thus presents an opportunity to integrate valuable links to phenotype into a genetic classification scheme.

To examine the performance of accessory genome information for phylogenetic reconstruction, we used the binary presence/absence data of the accessory genome computed for the set of six *L. monocytogenes *strains above, and constructed a MP tree (Figure 3). When comparing the tree topology of this accessory-based tree to that of the SNP-based core tree and the MLST tree, we found that the core- and accessory-based MP trees had an identical topology, but differed from the MLST-based tree in the placement of the two strains 10403S and F6900. Further studies will be required to determine the extent of the phylogenetic concordance between the core genome and the accessory genome among other bacterial groups.

#### The CAGF Module in the Examination of Pan-Genomic Differences

**Figure 4:**
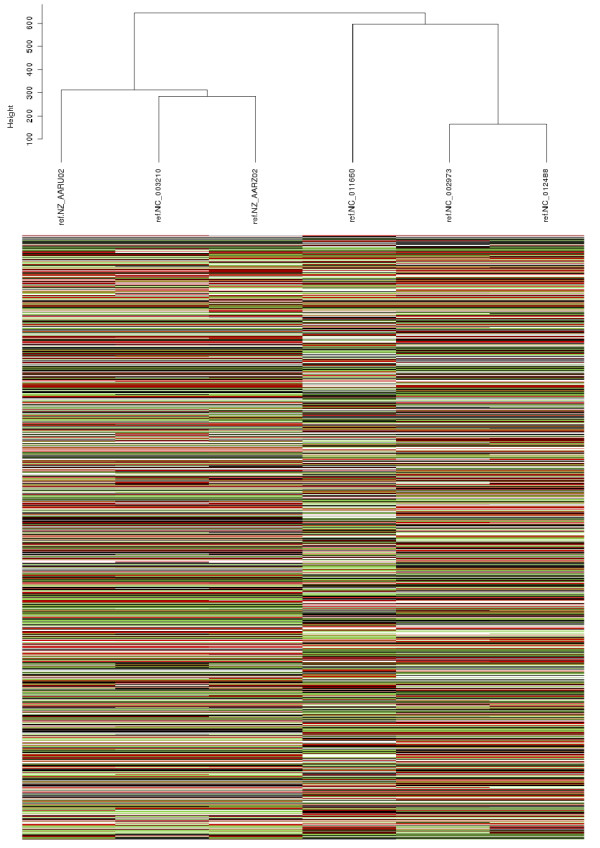
**Hierarchical clustering of SNPs among six *Listeria monocytogenes *strains**. Hierarchical clustering of the SNPs within the core genome of the *Listeria monocytogenes *strains F6900, 10403S, Clip81459, EGD-e, HCC23 and 4bF2365. The core genome was generated using the Panseq Core/Accessory Genome Analysis module with fragmentation size of 500 bp and sequence identity cutoff value of 85%. The dendrogram was produced by the statistical package R, using the hclust function with Euclidean distance and average linkage after substituting the ACTG character values with 0,1,2,3 respectively; black = "A", white = "T", red = "C" and green = "G".

**Figure 5:**
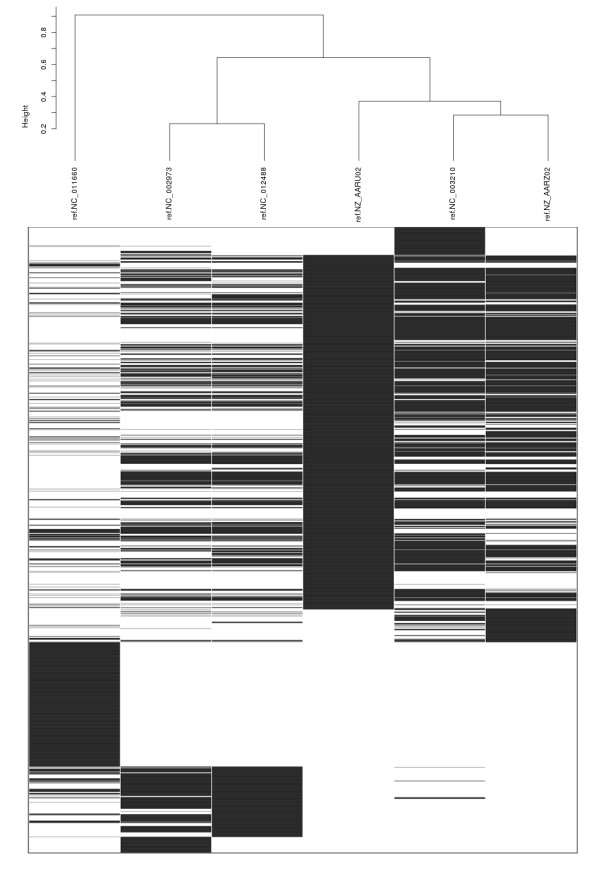
**Hierarchical clustering of accessory genome data among six *Listeria monocytogenes *strains**. Hierarchical clustering of the binary presence/absence values of the accessory genome of the *Listeria monocytogenes *strains F6900, 10403S, Clip81459, EGD-e, HCC23 and 4bF2365. The accessory genome was generated using the Panseq Core/Accessory Genome Analysis module with fragmentation size of 500 bp and sequence identity cutoff value of 85%. The dendrogram was produced by the statistical package R, using the hclust function with binary distance and average linkage; black indicates presence of a locus and white the absence of a locus.

As well as producing a concatenated core, the CAGF module also produces a table listing each SNP and its location and allele within each original sequence. This can be useful in examining individual differences or for hierarchical clustering of data. To demonstrate how the results of Panseq can be visualized, both tabular output files from the *Listeria *core/accessory analysis were used to create hierarchical clustering dendrograms: one based on the SNP character data from the core regions (Figure 4) and the other based on the binary presence/absence data of the accessory regions (Figure 5). Both dendrograms have the same tree topology, and the underlying data for both core and accessory regions shows only the differences among the genomic sequences, making comparisons between strains clearer than they might be from whole-genome comparisons where conserved as well as variable loci are considered in the analysis.

### (3) The Loci Selector (LS) Module

Molecular fingerprinting methods such as SNP analysis, multi-locus variable number tandem repeat analysis (MLVA) and comparative genomic fingerprinting (CGF) [29] often rely on a small number of loci to differentiate among a large number of bacterial strains. Determining which loci to use in a scheme requires selecting from, in some cases, thousands of loci. While manual inspection of a dataset is required to determine biologically relevant loci, Panseq provides an automated way to empirically determine the most variable and discriminatory loci from an investigator-defined set of variable character data which can range from SNPs to sequence presence/absence data.

#### The LS Module in the Comparison of Randomly Generated Data

The approach of the LS module in Panseq is to iteratively build the final loci set, including only the loci that produce the most unique fingerprints, and offer the most variability among input sequences at each step. This allows it to efficiently examine datasets that would be computationally prohibitive if all possible combinations were considered.

To test the Panseq LS module, we created a random binary dataset of 100 loci among 10 sequences in Microsoft Excel (Additional File 3). We subsequently ran Panseq with the 'best' option and found that 4 loci generated a unique profile for all 10 sequences, and that the four loci: locus84, locus40, locus79 and locus29 provided the maximum possible discrimination of 100 POD for the dataset. While these loci cannot be guaranteed to be the only four loci to generate the same results, they will always be one of the most discriminatory sets.

We then ran the same dataset through a Perl script that generated all possible 4-loci combinations, outputting those that produced 10 unique sequence profiles (Additional File 4). We found that 99132 unique combinations of 4 loci from the dataset yielded 10 unique sequence profiles. Evaluating every possible combination required 449 s on a computer running Ubuntu 9.10 with 3.6 GB of available RAM and AMD Phenom 8450 triple-core processors. Panseq was able to sort through the data to generate a single group of loci that contained not only the most unique profiles, but that provided the most discrimination among the input sequences, completing the task in one s.

#### The LS Module in the Analysis of SNP Genotyping Data

Any variable character data can be used as inputs in the LS module. To illustrate the functionality of the LS module, we used a set of 96 SNPs identified from *E. coli *O157:H7 by Manning et al. [47], for which the nucleotide value of each SNP was determined among a set of 17 *E. coli *O157:H7 genomic sequences [48]. This dataset was first modified to represent any unknown character with the '?' symbol, and to replace any locus described by two characters with a single character (Additional File 5).

This dataset was analyzed by the LS module to give the 20 best loci from the 96 available. The results are presented in Table 3, and show that the first locus selected by Panseq was ECs2696, a locus with three alleles and therefore more initial fingerprints than any other locus except ECs2006, which also had three alleles among the strains. However, ECs2696 provided more POD among the strains than ECs2006 and was thus selected by Panseq as the initial locus. The eighth locus added (ECs5359) was the last to provide unique strain sequence profiles. This locus differentiated the K-12 strain and *E. coli *O157:H7 strains EC508 and EC71074. Every subsequent locus (9-20) was chosen by the program for its ability to offer discrimination among the remaining strain pairs, while ensuring that highly variable loci that contain very similar allele patterns among strains (i.e are not informative) did not replace loci in the set that offered discrimination among fewer, but nevertheless diverse strains.

**Table 3:** The 20 best loci as chosen by the LS module of Panseq from the original 96 loci of Additional File 5.

Locus	TW14588	Sakai	EDL933	EC4501	EC4486	EC4401	EC4206	EC4196	EC4115	EC4113	EC4076	EC4045	EC4042	EC4024	EC869	EC508	EC71074	EC33264	K12	POD
ECs2696	C	C	G	C	A	A	A	A	A	A	A	A	A	A	A	A	A	A	A	63
ECs2775	T	G	G	G	G	G	G	G	G	G	G	G	T	G	G	T	G	-	-	42
ECs2375	C	C	C	C	T	T	T	T	T	T	T	T	T	T	C	C	C	C	C	90
ECs4067	A	C	A	A	A	A	C	A	A	A	A	A	A	A	A	A	A	A	A	34
ECs1262	T	T	T	T	C	C	C	C	C	C	C	C	C	C	T	C	C	T	-	72
ECs1272	T	T	T	T	A	A	A	A	A	A	A	A	A	A	T	A	A	A	-	65
ECs1860	G	G	G	G	G	G	G	G	G	G	G	G	G	T	G	G	G	G	G	18
ECs5359	T	T	T	T	T	T	T	T	T	T	T	T	T	T	T	T	T	-	G	17
ECs3830	C	C	C	C	T	T	T	T	T	T	T	T	T	T	C	C	C	C	C	90
ECs2357	C	C	C	C	A	A	A	A	A	A	A	A	A	A	C	A	A	C	-	72
ECs4022	G	G	G	G	A	A	A	A	A	A	A	A	A	A	G	A	A	-	G	72
ECs0593	T	T	T	T	C	C	C	C	C	C	C	C	T	C	C	C	C	C	C	70
ECs4380	G	G	G	G	A	A	G	A	A	A	A	A	A	A	A	A	A	A	-	65
ECs0606	A	A	C	A	C	C	C	C	C	C	C	C	C	C	A	C	C	-	-	52
ECs2514	T	T	C	T	T	T	T	T	T	T	T	T	T	T	T	T	T	C	C	48
ECs2006	G	G	C	G	G	G	G	G	G	G	G	G	G	G	G	G	A	-	-	31
ECs2852	C	T	C	C	C	C	C	C	C	C	C	C	C	C	C	C	C	C	C	18
ECs4251	G	G	G	G	A	A	A	A	A	A	A	A	A	A	G	G	G	G	G	90
ECs4305	A	A	A	A	C	C	C	C	C	C	C	C	C	C	C	C	C	C	C	60
ECs4479	G	G	G	G	T	T	T	T	T	T	T	T	T	T	G	T	T	-	-	60

Loci are listed in order of choice and with points of discrimination (POD).

#### Advantages of Panseq over Other Related Programs

While many sequence analysis programs exists, some with overlapping capabilities, no two are identical with respect to the tasks they perform. With enough time and knowledge one can parse the output of a sequence alignment program such as BLAST or MAUVE manually, but there is a considerable time savings and ease of use with a program such as Panseq that automates the process. Panseq is unique in its single-step novel region finding options among groups and specific to individual sequences, which other *in silico *subtractive programs such as mGenomeSubtractor [22] and nWayComp [23] lack. Panseq also provides a comprehensive analysis of the pan-genome, automatically generating analyses that can only be partially accomplished by any other single program; eg. With MAUVE [49] a list of SNPs can be generated and a display of the similarities/differences between the genomes is produced, but the underlying nucleotide sequence is not automatically extracted. With Panseq, the underlying sequence data is automatically extracted, the segments compared among all sequences and presented in tabular form and input files for phylogenetic programs are automatically created. Further, the SNP table or binary presence/absence table of the accessory genome can be used directly in the LS module, for the selection of the most discriminatory loci.

## Conclusion

We have developed Panseq, a freely available online program to quickly find and extract strain- or group-specific novel accessory genomic information as well as the complete pan-genome for a group of genomic sequences. Panseq produces alignments of the core genome of each sequence and determines the distribution of accessory regions among all sequences analyzed. Panseq makes use of the MUMmer alignment algorithm for whole genome comparisons and the BLASTn algorithm for local sequence comparisons and can efficiently compute values for large numbers of sequences. Additionally, Panseq is able to rapidly identify the most variable and discriminatory loci set in an iterative manner from single character tabular data.

## Availability and Requirements

**Project name**: Panseq

**Project home page**: http://76.70.11.198/panseq

**Operating system(s)**: Platform independent

**Programming language**: Perl

**Other requirements**: Firefox 2.0+, Internet Explorer 6.0+, Google Chrome or compatible web-browser

**License**: Freely available

## Authors' contributions

CRL planned the project, wrote and tested the Panseq code and wrote the manuscript; CB performed the experimental work; ENT contributed to revision of the manuscript and with the testing of Panseq; YZ contributed to revision of the manuscript and with the testing of Panseq; AK and AV implemented Panseq on the server and contributed to revision of the manuscript; JET contributed to project planning, revision of the manuscript and the testing of Panseq; VPJG led the project planning, revision of the manuscript and the testing of Panseq. All authors have read and approved the final manuscript.

## Supplementary Material

Additional file 1 **Comparison of Sequence Analysis Programs**. A feature comparison of currently available web-servers and standalone sequence analysis programs. Click here for file

Additional file 2 **Genomic Island Comparison**. An island-by-island comparison of Panseq to previously reported *E. coli *K12 and *E. coli *O157:H7 EDL933 genomic islands. Click here for file

Additional file 3 **Random Binary Data File**. A table of randomly generated binary digits, simulating the presence/absence of 100 loci among 10 sequences. Click here for file

Additional file 4 **Exhaustive Four Loci Combinations**. All four-loci combinations of the data from Additional File 3 generating the maximum 10 unique fingerprints. Click here for file

Additional file 5 **SNP Genotyping Data**. A set of 96 SNPs identified from *E. coli *O157:H7 by Manning et al. [47], for which the nucleotide value of each SNP was previously determined among a set of 17 *E. coli *O157:H7 genomic sequences [48]. Click here for file
